# Aminomethylphosphonic Acid and Methoxyacetic Acid Induce Apoptosis in Prostate Cancer Cells

**DOI:** 10.3390/ijms160511750

**Published:** 2015-05-22

**Authors:** Keshab R. Parajuli, Qiuyang Zhang, Sen Liu, Zongbing You

**Affiliations:** Departments of Structural & Cellular Biology and Orthopaedic Surgery, Tulane Cancer Center and Louisiana Cancer Research Consortium, Tulane Center for Stem Cell Research and Regenerative Medicine, Tulane Center for Aging, Tulane University, New Orleans, LA 70112, USA; E-Mails: kparajuli55@gmail.com (K.R.P.); qzhang3@tulane.edu (Q.Z.); senliu@yahoo.com (S.L.)

**Keywords:** prostate cancer cells, cell death, apoptosis, AMPA, MAA

## Abstract

Aminomethylphosphonic acid (AMPA) and its parent compound herbicide glyphosate are analogs to glycine, which have been reported to inhibit proliferation and promote apoptosis of cancer cells, but not normal cells. Methoxyacetic acid (MAA) is the active metabolite of ester phthalates widely used in industry as gelling, viscosity and stabilizer; its exposure is associated with developmental and reproductive toxicities in both rodents and humans. MAA has been reported to suppress prostate cancer cell growth by inducing growth arrest and apoptosis. However, it is unknown whether AMPA and MAA can inhibit cancer cell growth. In this study, we found that AMPA and MAA inhibited cell growth in prostate cancer cell lines (LNCaP, C4-2B, PC-3 and DU-145) through induction of apoptosis and cell cycle arrest at the G1 phase. Importantly, the AMPA-induced apoptosis was potentiated with the addition of MAA, which was due to downregulation of the anti-apoptotic gene baculoviral inhibitor of apoptosis protein repeat containing 2 (BIRC2), leading to activation of caspases 7 and 3. These results demonstrate that the combination of AMPA and MAA can promote the apoptosis of prostate cancer cells, suggesting that they can be used as potential therapeutic drugs in the treatment of prostate cancer.

## 1. Introduction

Aminomethylphosphonic acid (AMPA; linear chemical formula: CH6NO3P) is the primary degradation product of glyphosate (*N*-(phosphonomethyl)glycine), which is a broad-spectrum systemic herbicide used to kill weeds, especially annual broadleaf weeds and grasses known to compete against commercial crops grown around the globe [1]. In the environment, glyphosate can be naturally converted into AMPA [2]. AMPA has no significant toxicity in acute, subchronic and chronic animal studies, nor any genotoxicity, teratogenicity or carcinogenicity [3,4]. When AMPA was administered via gavage at a dose of 6.7 mg/kg in rats, approximately 20% of the AMPA was absorbed, and 74% of the administered dose was excreted in the feces over a five-day period of experimental observation. The absorbed AMPA was not bio-transformed and was excreted rapidly in the urine with approximately 65% of the absorbed dose eliminated in the urine within 12 h and essentially 100% excreted between 24 and 120 h. Only trace residues were detected in various organs, including liver, kidney and skeletal muscle, five days after dosing [3,4]. AMPA and glyphosate are analogs to glycine. Recently, glycine was revealed to play a key role in rapid cancer cell proliferation [5]. In rapidly-proliferating cancer cells, there is an increased reliance on glycine metabolism, a phenotype that was not observed in rapidly-proliferating non-transformed cells [5]. Glycine metabolism may therefore represent a metabolic vulnerability in rapidly-proliferating cancer cells that may be targeted for therapeutic benefits. As analogs to glycine, glyphosate and AMPA were also found to inhibit proliferation and promote apoptosis in cancer cells, but not in normal cells in our previous study [6]. However, higher concentrations of this chemical may affect normal cells and produce adverse side effects. One of the strategies to improve AMPA’s inhibitory actions on cancer cells and to reduce its side effects is to find a compound that can potentiate AMPA’s efficacy, thus reducing its dosage.

Methoxyacetic acid (MAA) is the primary active metabolite of ester phthalates widely used in industry as gelling, viscosity and stabilizer reagents [7]. MAA exposure is associated with various developmental and reproductive toxicities in both rodents and humans, including neural toxicity, blood and immune disorders, limb degeneration and testicular toxicity [8,9,10]. The mechanisms of MAA-induced toxicities are multiple. MAA induces the production of reactive oxygen species, resulting in DNA damage and loss of mitochondrial membrane potential in normal human fibroblasts [7]. MAA treatment alters the expression of androgen receptor (AR) and androgen-binding protein (ABP) in a stage-specific manner in rat seminiferous tubules [11]. MAA treatment downregulates the expression of estrogen receptor α (ERα) and estradiol-induced gene expression in human breast cancer cell line MCF-7 and mouse uterus [12], but increases ERβ expression by inducing apoptosis in pachytene spermatocytes in rats [11]. MAA has been found to activate the tyrosine kinase-PI3K pathway and other pathways to enhance or antagonize androgen-induced gene expression [9,10,13]. Similarly, MAA can activate mitogen-activated protein kinase (MAPK) and inhibit histone deacetylases (HDACs), thus increasing the levels of acetylated histone H4, like the other well-known HDAC inhibitors, such as trichostatin, valproic acid and butyric acid [14]. In fact, it has been reported that MAA-induced hyperacetylation of histone H3 and H4 is associated with rapid spermatocyte death [15]. Some HDAC inhibitors (suberanilohydroxamic acid and romidepsin) have been approved for the treatment of cutaneous T-cell lymphoma, while panobinostat and valproic acid are being tested in the treatment of prostate cancer, breast cancer, cervical cancer, ovarian cancer and lymphomas [16]. We have previously shown that MAA can induce apoptosis and growth arrest of prostate cancer cells [17].

Our rationale was that intracellular glycine is converted from serine, which may be inhibited by AMPA (a glycine analog) [18], and glycine is also converted from sarcosine, which may be inhibited by MAA (a sarcosine analog) [19]. Therefore, we speculated that a combination of AMPA and MAA might inhibit glycine synthesis more effectively, hence potentiating the inhibition of prostate cancer growth. In the present study, we tested the effects of a combination of AMPA and MAA on two immortalized human normal prostatic epithelial cell lines (RWPE-1 and pRNS-1-1) and four human prostate cancer cell lines (LNCaP, C4-2B, PC-3 and DU-145). We found that the combination of AMPA and MAA significantly induced the apoptosis and growth arrest of prostate cancer cells. The apoptosis induced by the combination of AMPA and MAA was highly associated with decreased protein expression of baculoviral inhibitor of apoptosis protein repeat containing 2 (BIRC2), whereas the G1 arrest caused by the combination of AMPA and MAA was closely associated with induction of p21 and reduction of cyclin D3. BIRC2 is also named cellular inhibitor of apoptosis protein (cIAP) 1 [20]. cIAP1 and cIAP2 have an *N*-terminal BIRC domain and a *C*-terminal ring domain that confers E3 ubiquitin ligase activity. CIAP2 also contains a caspase recruitment domain (CARD), which is involved in auto-inhibition of its E3 ligase activity [21]. It is known that BIRC2 inhibits caspases 7 and 3 [22]. Therefore, decreased cIAP1 leads to activation of caspases 7 and 3, thus inducing apoptosis. Our findings suggest that MAA can potentiate AMPA’s anti-cancer activities by inhibiting anti-apoptotic protein and activating pro-apoptotic proteins.

## 2. Results and Discussion

### 2.1. The Aminomethylphosphonic Acid (AMPA) and Methoxyacetic Acid (MAA) Combination Inhibits Prostate Cancer Cell Viability

**Figure 1:**
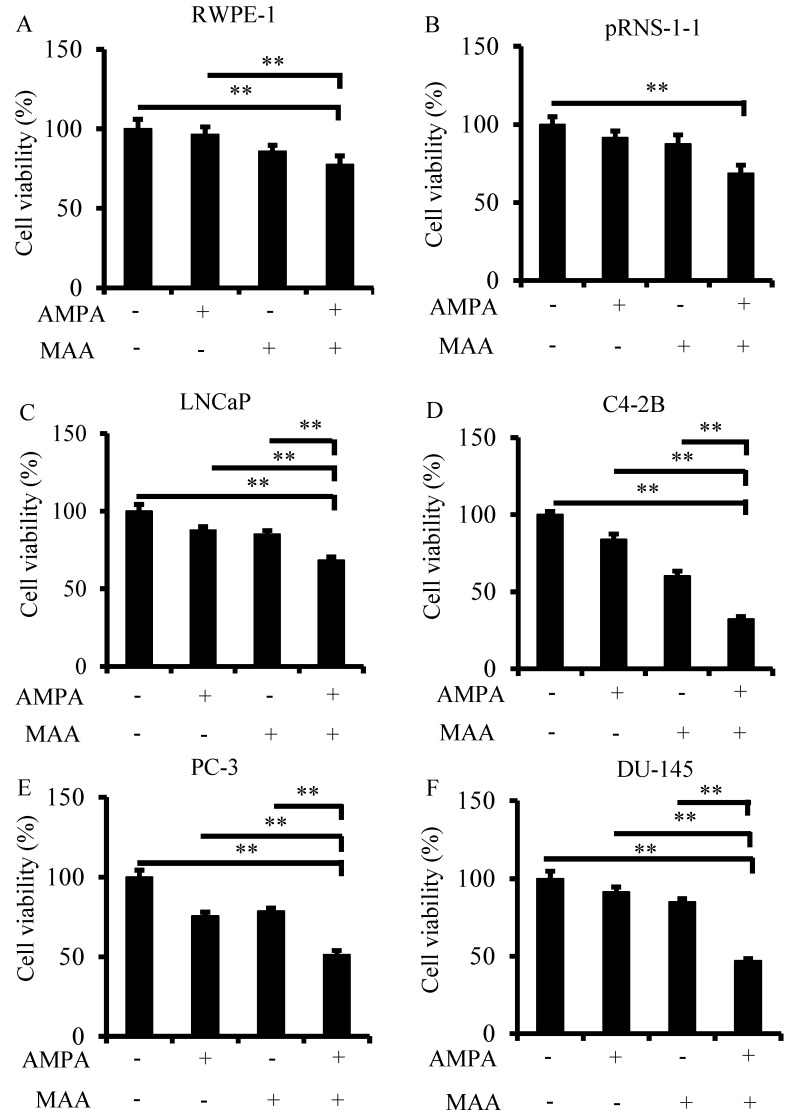
The combination of aminomethylphosphonic acid (AMPA) and methoxyacetic acid (MAA) suppresses cancer cell viability. (**A**–**F**) Normal prostate epithelial cells and prostate cancer cells were plated in 96-well plates in triplicate per group and then treated with 15 mM AMPA, 5 mM MAA and a combination of both for 72 h. The viable cells were measured using the CellTiter-Glo^®^ Luminescent Cell Viability Assay. The data are presented as the mean ± standard error of the mean (SEM) of three independent experiments (*n* = 3). ** *p* < 0.01.

Therapy for advanced prostate cancer centers on suppressing systemic androgens and blocking activation of the androgen receptor. However, nearly all patients develop castration-resistant prostate cancer (CRPC). The currently available treatments for CRPC can only extend patient’s survival by 2.4 to 4.8 months [23]. Thus, new therapeutics is urgently needed for this type of malignancy. Based on our previous study, AMPA and MAA both have inhibitory effects on prostate cancer cells. In order to enhance AMPA’s efficacy and reduce its dosage, we investigated the effects of a combination of AMPA and MAA on the growth of prostate cancer cells. Our previous studies showed that 50 mM AMPA and 20 mM MAA can significantly inhibit the growth of prostate cancer cells individually [6,17]. In the present study, we used 15 mM AMPA and 5 mM MAA to treat the two immortalized human normal prostatic epithelial cell lines (RWPE-1 and pRNS-1-1) and four prostate cancer cell lines (LNCaP, C4-2B, PC-3 and DU-145) alone or in combination. We found that the number of viable cells was decreased by approximately 9% to 24% or 15% to 40% in the four prostate cancer cells compared to 4% to 9% or 12% to 14% in normal prostatic epithelial cells, respectively, when treated with AMPA or MAA alone. However, the number of viable cells decreased by 32% to 68% in the four prostate cancer cells, compared to 22% and 31% in RWPE-1 and pRNS-1-1 cells when treated with the combination of AMPA and MAA (Figure 1A–F). Thus, MAA obviously potentiates the effects of AMPA, especially on the rapidly proliferating prostate cancer cells, but less so on the normal prostatic cells. At these dosages, AMPA has little effect on normal prostatic cells. Although MAA has been reported to be a testicular toxicant in mammals [24,25], this toxicity would be acceptable to most of the prostate cancer patients, since the majority of the patients are old and have passed their reproductive age [15]. Therefore, AMPA and MAA combination appears to be a promising therapy in the treatment of prostate cancer.

### 2.2. The Combination of AMPA and MAA Potentiates Apoptosis in Prostate Cancer Cells

**Figure 2:**
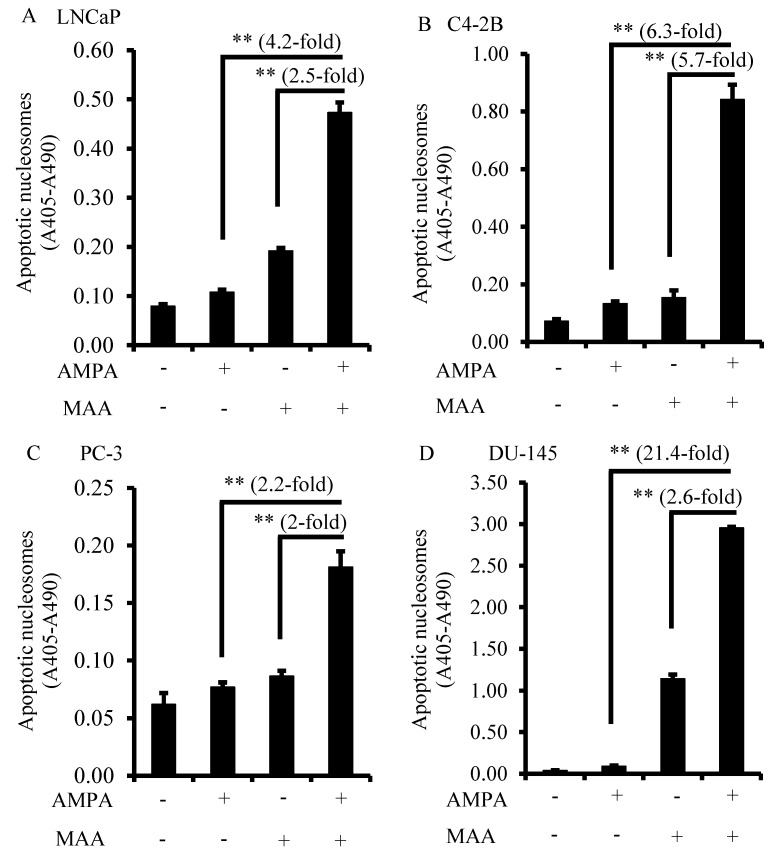
The AMPA and MAA combination induces apoptosis in prostate cancer cells. (**A**–**D**) Prostate cancer cells were plated in 12-well plates in triplicate per group and treated with 15 mM AMPA, 5 mM MAA and a combination of AMPA and MAA for 24 h. Apoptotic nucleosomes were measured using the Cell Death Detection ELISA kit. Apoptotic nucleosomes were calculated by absorbance at 405 nm (A405) minus absorbance at 490 nm (A490). The data are presented as the mean ± SEM of three independent experiments (*n* = 3). ** *p* < 0.01.

To know why the combination of AMPA and MAA can inhibit prostate cancer cell growth, we measured the apoptotic nucleosomes in the cells treated with 15 mM AMPA and 5 mM MAA, either alone or in combination for 24 h. Although the induced apoptotic nucleosomes were slightly increased when treated with AMPA or MAA alone compared to the non-treated cells, the combination of AMPA and MAA increased the apoptotic nucleosomes by 4.2- and 2.5-fold in LNCaP cells, by 6.3- and 5.7-fold in C4-2B cells, by 2.1- and two-fold in PC3 cells and by 21.4- and 2.6-fold in DU-145 cells, compared to the treatment with AMPA or MAA alone (Figure 2A–D). These results indicated that AMPA and MAA at low concentrations potentiate the apoptosis of prostate cancer cells.

### 2.3. The Combination of AMPA and MAA Blocks the Entry of Cells from the G1 to S Phase of the Cell Cycle

**Figure 3:**
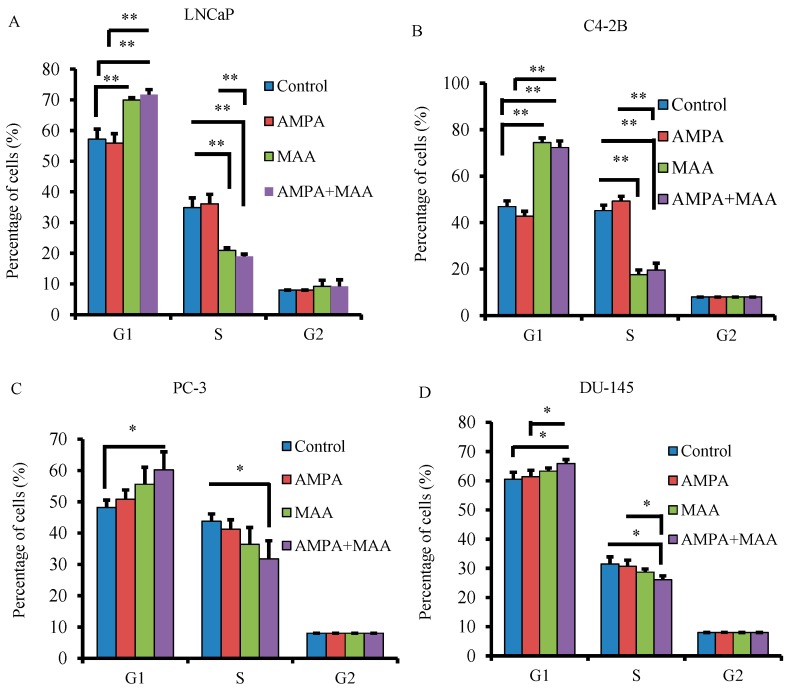
AMPA and MAA block the G1/S transition of the prostate cancer cell cycle. (**A**–**D**) Prostate cancer cells were plated in 60-mm dishes in triplicate per group and treated with 15 mM AMPA, 5 mM MAA, alone or in combination, for 24 h. The control groups was treated with phosphate-buffer saline (PBS). The percentages of cells at G1 (and G_0_), S and G2 (and M) phases were determined by flow cytometry analysis. The data are presented as the mean ± SEM of three independent experiments (*n* = 3). * *p* < 0.05; ** *p* < 0.01.

To determine if the combination of AMPA and MAA induces cell cycle arrest, we treated four types of prostate cancer cells for 24 h and analyzed the percentage of cells in the G1 (and G0), S and G2 (and M) phase of the cell cycle using flow cytometry analysis. We found that MAA alone increased the percentage of LNCaP and C4-2B cells at the G1/G0 phase and decreased the percentage of cells at the S phase (Figure 3A,B; *p* < 0.01), whereas MAA alone did not have significant effects in PC-3 and DU-145 cells (Figure 3C,D; *p* > 0.05). However, the combination of AMPA and MAA significantly increased the percentage of PC-3 and DU-145 cells at the G1/G0 phase and decreased the percentage of cells at the S phase, whereas the number of cells in the G2/M phase was not affected (Figure 3C,D; *p* < 0.05). In addition, there were not any significant differences in all four cell lines when treated with AMPA alone (Figure 3A–D; *p* > 0.05). These results indicated that the combination of AMPA and MAA blocks the G1/S transition in PC-3 and DU-145 cell lines. Our previous study demonstrated that AMPA at 50 mM can arrest cancer cells in the G1/G0 phase of the cell cycle, thus inhibiting entry into the S phase [6]. MAA has also been demonstrated to be an HDAC inhibitor [14,15], which suppresses the growth of four prostate cancer cell lines (LNCaP, C4-2B, PC-3 and DU-145) in a dose-dependent manner by inducing apoptosis and G1 arrest.

### 2.4. The AMPA and MAA Combination Induces Changes in the Expression Levels of Genes Involved in the Cell Cycle and Apoptosis

**Figure 4:**
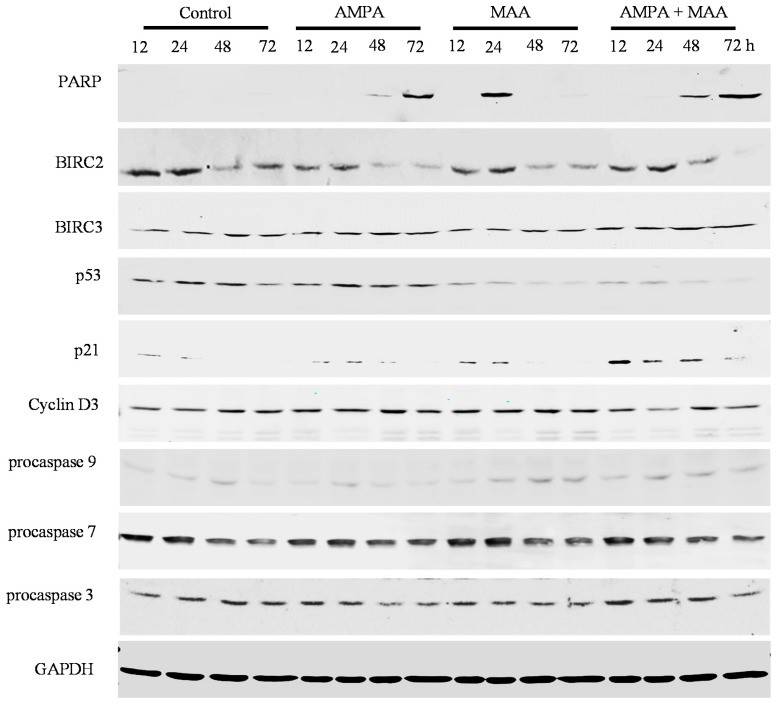
AMPA and MAA induce changes in the expression levels of genes involved in cell cycle arrest and apoptosis. LNCaP cells were exposed to 15 mM AMPA, 5 mM MAA or a combination of both AMPA and MAA for different time periods. The protein extracts were analyzed by Western blot to detect the indicated proteins. Glyceraldehyde 3-phosphate dehydrogenase (GAPDH) was probed as the loading control.

**Figure 5:**
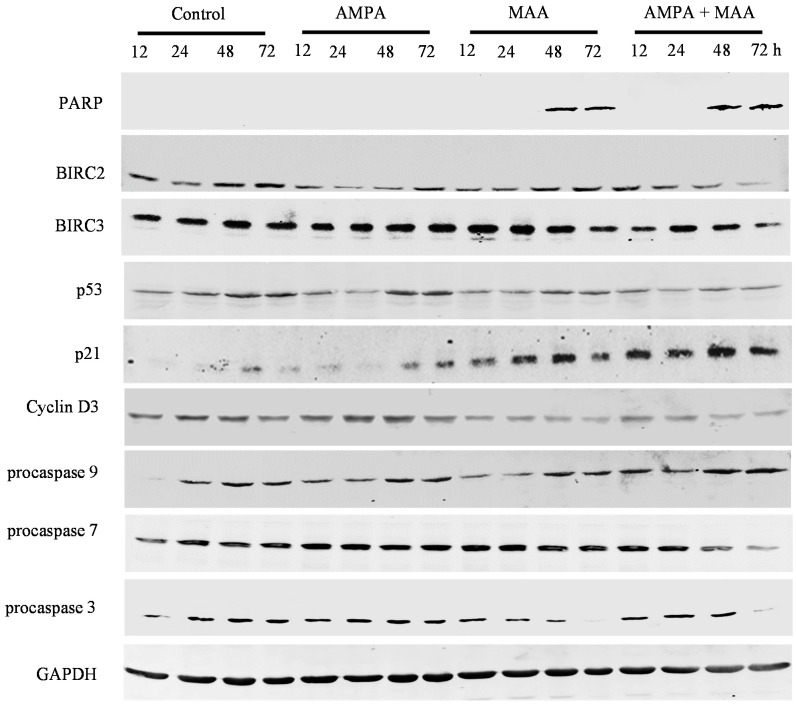
AMPA and MAA induce changes in the expression levels of genes involved in cell cycle arrest and apoptosis. C4-2B cells were exposed to 15 mM AMPA, 5 mM MAA or a combination of both AMPA and MAA for different time periods. The protein extracts were analyzed by Western blot to detect the indicated proteins. GAPDH was probed as the loading control.

**Figure 6:**
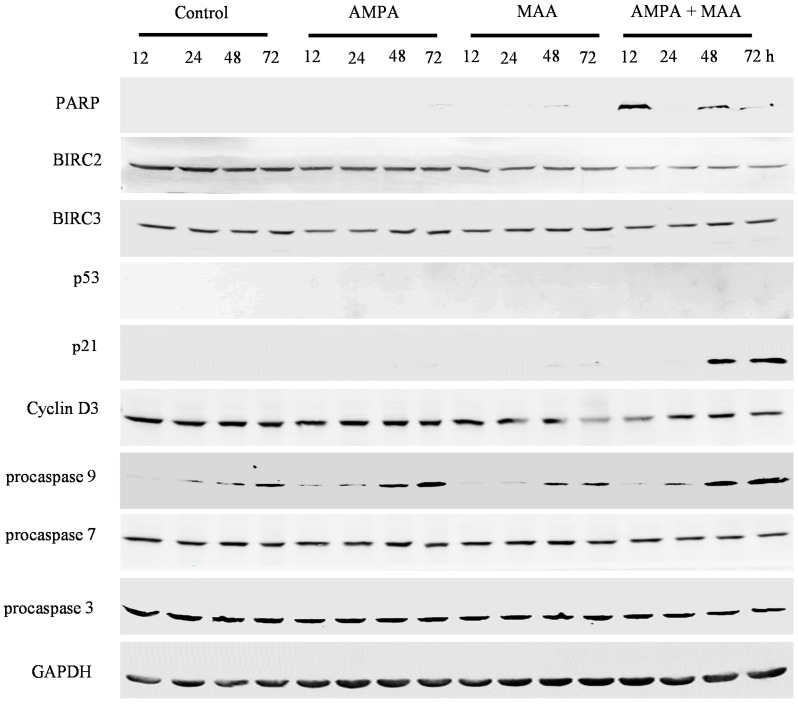
AMPA and MAA induce changes in the expression levels of genes involved in cell cycle arrest and apoptosis. PC-3 cells were exposed to 15 mM AMPA, 5 mM MAA or a combination of both AMPA and MAA for different time periods. The protein extracts were analyzed by Western blot to detect the indicated proteins. GAPDH was probed as the loading control.

**Figure 7:**
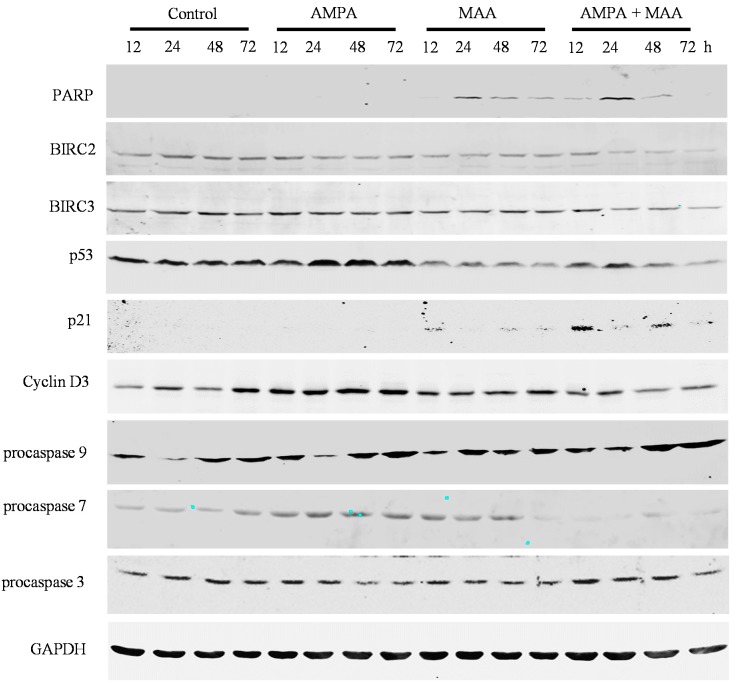
AMPA and MAA induce changes in the expression levels of genes involved in cell cycle arrest and apoptosis. DU-145 cells were exposed to 15 mM AMPA, 5 mM MAA or a combination of both AMPA and MAA for different time periods. The protein extracts were analyzed by Western blot to detect the indicated proteins. GAPDH was probed as the loading control.

To study the genes involved in cell cycle arrest and apoptosis in prostate cancer cells treated with the combination of AMPA and MAA, we did Western blot analysis of the protein expression. We found that the combination treatment clearly increased the levels of cleaved poly(ADP-ribose) polymerase (PARP) in C4-2B, PC-3 and DU-145 cell lines in a time-dependent manner compared to the cells treated with AMPA or MAA alone, though there was no obvious increase in the LNCaP cell line (Figure 4, Figure 5, Figure 6 and Figure 7). PARP cleavage has been widely used as an indicator of apoptosis marker [26,27]. This finding confirmed that the combination of AMPA and MAA induced apoptosis in prostate cancer cells.

We found that the combination treatment clearly decreased the protein levels of BIRC2 in all four prostate cancer cell lines and decreased BIRC3 levels in C4-2B and DU-145 cells at late time points (48 and 72 h) (Figure 4, Figure 5, Figure 6 and Figure 7). The protein level of BIRC2 was decreased more obviously than that of BIRC3, which is another member of the IAP family [28]. It has been shown that proteasome-mediated degradation of BIRC2 can relieve the inhibitory function of BIRC2 on caspases, thus activating caspase-mediated apoptosis [29,30]. We found that the combination of AMPA and MAA increased the levels of procaspase 9 starting from 24 or 48 h in C4-2B, PC-3 and DU-145 cell lines (Figure 5, Figure 6 and Figure 7). In contrast, the combination of AMPA and MAA decreased the levels of procaspases 7 and 3 at different time points (12, 24, 48 and 72 h) (Figure 4, Figure 5, Figure 6 and Figure 7). The increases of procaspase 9 levels and simultaneously decreases of procaspase 3 levels induced by the combination of AMPA and MAA may mediate apoptosis, as shown in a previous study [31]. The decrease of procaspase 3 indicated cleavage of the proenzyme and activation of caspase 3, which is a key executioner caspase [32]. The previous study demonstrated that MAA induces apoptosis of rat germ cells through the release of mitochondrial cytochrome c, which further activates caspases 9 and 3 [33]. BIRC2 has also been reported to be able to bind to caspases 7 and 9, thus serving as a weak inhibitor of caspases 9, 7 and 3 [34]. Later on, it was found that BIRC2 potently inhibited activation of procaspase 3 [35]. Thus, the decreased levels of procaspases 7 and 3 may be due to down-regulation of BIRC2.

We further checked the protein levels of p53 and its downstream gene p21. We found that the combination of AMPA and MAA obviously increased the levels of p21 at 12 h after treatment in LNCaP, C4-2B and DU-145 cells (Figure 4, Figure 5 and Figure 7) and at a later time point in PC-3 cells (Figure 6). However, the levels of p53 protein were decreased slightly in the cells upon the combination treatment. Therefore, the induced expression of p21 in the cells under the combination treatment is independent of p53 protein level. This result is consistent with the previous study showing that MAA induces p21 transcription through inhibition of HDAC activities, in a p53 family-independent manner [17]. In addition, the protein levels of cyclin D3 were decreased in the cells under the combination treatment compared to the cells treated with AMPA or MAA alone (Figure 4, Figure 5, Figure 6 and Figure 7). The downregulated cyclin D3 may contribute to the cell cycle inhibition [32]. The combination of AMPA and MAA increased p21 and decreased the protein level of cyclin D3, thus inducing G1 arrest.

It is noted that the combination of AMPA and MAA appears to be more effective in the inhibition of PC-3 and DU-145 growth than LNCaP growth (Figure 1). PC-3 and DU-145 cells do not express intracellular androgen receptor (AR) [36,37], while all three cell lines express membrane-associated AR [38,39,40]. Activation of the membrane-associated AR may potentiate the anti-proliferative effects of chemotherapeutics [41,42,43]. Given that MAA alters AR expression in rat seminiferous tubules [11], it is an intriguing question whether MAA acts through affecting AR expression in these cell lines. MAA has been shown to potentiate AR signaling even in the presence of AR antagonists, such as bicalutamide [9]. It may be possible that such a potentiation of AR signaling in LNCaP cells slightly affects MAA’s inhibitory function in LNCaP cells, compared to PC-3 and DU-145 cells that lack intracellular AR.

Taken together, the combination of AMPA and MAA at low doses significantly induced apoptosis and, to a lesser extent, cell cycle arrest, in four prostate cancer cell lines in the present *in vitro* study. These findings suggest that a further *in vivo* study is warranted to test if the combined AMPA and MAA treatment may efficaciously inhibit prostate tumor growth in animals. Based on the present *in vitro* study, it appears that the combined AMPA and MAA treatment may be of potential in the treatment of prostate cancer.

## 3. Experimental Section

### 3.1. Cell Culture

The sources and cell culture conditions of two immortalized human normal prostatic epithelial cell lines (RWPE-1 and pRNS-1-1) and four human prostate cancer cell lines (LNCaP, C4-2B, PC-3 and DU-145) were described previously [6]. Cells were cultured in a 5% CO_2_ humidified incubator at 37 °C.

### 3.2. Cell Viability Assay

The number of live cells was determined using the CellTiter-Glo^®^ Luminescent Cell Viability Assay (Promega Corp, Fitchburg, WI, USA) as described previously [6]. Cell viability was calculated as (luminescence of the treatment group − background luminescence) ÷ (luminescence of the control group − background luminescence) × 100%. The data are presented as the mean and standard error of the mean (SEM) of three independent experiments.

### 3.3. Detection of Apoptotic Nucleosomes

Cells were seeded on 12-well plates with 1 × 10^5^ cells/well in triplicate per group in the complete culture medium with fetal bovine serum (FBS). After overnight incubation, cells were treated with 15 mM AMPA, 5 mM MAA and the AMPA and MAA combination for 24 h; a control group was treated with phosphate-buffered saline (PBS). Apoptotic nucleosomes were detected using the Cell Death Detection ELISA kit (Roche Diagnostics Corporation, Indianapolis, IN, USA) according to the manufacturer’s instructions [44]. Absorbance was measured at 405 nm (A405) with a reference wavelength at 490 nm (A490) using a plate reader (Bio-Tek U.S., Winooski, VT, USA). The amount of apoptotic nucleosomes was represented by A405−A490.

### 3.4. Cell Cycle Analysis

Cells were treated without or with 15 mM AMPA or 5 mM MAA or AMPA and MAA combination for 24 h. The percentage of cells at G1/G0, S and G2/M phases was determined by flow cytometry analysis as described previously [6].

### 3.5. Western Blot Analysis

Cells were treated without or with 15 mM AMPA, 5 mM MAA or the AMPA and MAA combination for 0, 12, 24, 48 and 72 h. Proteins were extracted for Western blot analysis as described previously [6]. Rabbit anti-caspase 9, rabbit anti-caspase 7 and mouse anti-cyclin D3 antibodies were purchased from Cell Signaling Technology (Danvers, MA, USA). Mouse anti-p53, rabbit anti-BIRC2 and rabbit anti-BIRC3 antibodies were obtained from Santa Cruz Biotechnology (Dallas, TX, USA). Mouse anti-GAPDH, mouse anti-caspase 3 and rabbit anti-cleaved poly(ADP-ribose) polymerase (PARP) antibodies were purchased from EMD Millipore Corp (Bilerica, MA, USA). Rabbit anti-p21 antibody was bought from Abcam, Cambridge, MA, USA.

### 3.6. Statistical Analysis

Results from this study were presented as the mean ± SEM. Statistical analysis was performed using two-tailed Student’s *t-*test. A *p*-value <0.05 was considered statistically significant.

## 4. Conclusions

The present study demonstrated that a combination of AMPA and MAA can inhibit prostate cancer cell growth through inducing apoptosis and cell cycle arrest. Induction of apoptosis may be due to downregulation of BIRC2 (cIAP1), leading to activation of pro-apoptotic factors, such as caspases 7 and 3. Induction of cell cycle arrest may be due to up-regulation of p21 expression at the early time and downregulation of cyclin D3 expression at the late time. These findings suggest that the AMPA and MAA combination may have potential in the treatment of prostate cancer.
